# Dose-dependent effects of lesogaberan on reflux measures in patients with refractory gastroesophageal reflux disease: a randomized, placebo-controlled study

**DOI:** 10.1186/1471-230X-14-188

**Published:** 2014-11-18

**Authors:** Philip B Miner, Debra G Silberg, Magnus Ruth, Frank Miller, John Pandolfino

**Affiliations:** Oklahoma Foundation for Digestive Research, 535 NW 9th Street, Suite 325, Oklahoma City, OK USA; Clinical Development, AstraZeneca LP, Wilmington, DE USA; Research and Development, AstraZeneca, Mölndal, Sweden; Statistics & Informatics, AstraZeneca, Södertälje, Sweden; Department of Medicine, Northwestern University, Chicago, IL USA

**Keywords:** Gastroesophageal reflux, Lower esophageal sphincter, GABA-B agonist, Dose–response, Clinical study

## Abstract

**Background:**

The γ-aminobutyric acid type B-receptor agonist lesogaberan (AZD3355) has been developed for use in patients with gastroesophageal reflux disease (GERD) symptoms despite proton pump inhibitor (PPI) therapy (partial responders). This study aimed to explore the dose–response effect of lesogaberan on reflux episodes in partial responders.

**Methods:**

In this randomized, single-centre, double-blind, crossover, placebo-controlled study, partial responders taking optimised PPI therapy were given 30, 90, 120 and 240 mg doses of lesogaberan. Each dose was given twice (12 h apart) during a 24-h period, during which impedance–pH measurements were taken.

**Results:**

Twenty-five patients were included in the efficacy analysis and 27 in the safety analysis. The effect of lesogaberan on the mean number of reflux episodes was dose-dependent, and all doses significantly reduced the mean number of reflux episodes relative to placebo. Lesogaberan also dose-dependently reduced the mean number of acid reflux episodes (except the 30 mg dose) and weakly acid reflux episodes (all doses) significantly, relative to placebo. Regardless of dose, lesogaberan had a similar effect on the percentage of time with esophageal pH < 4 [mean reduction: 68.5% (30 mg), 54.2% (90 mg), 65.9% (120 mg), 72.1% (240 mg); p < 0.05 except 90 mg dose]. No adverse events led to discontinuation and no serious adverse events occurred during active treatment.

**Conclusions:**

Lesogaberan inhibited reflux in a dose-dependent manner in partial responders taking optimised PPI therapy, and these effects were significant versus placebo. All lesogaberan doses were well tolerated and were not associated with clinically relevant adverse events.

**Trial registration:**

ClinicalTrials.gov identifier: NCT01043185.

## Background

Gastroesophageal reflux disease (GERD) is widespread, affecting approximately 10–20% of people in Europe and North America [1]. The primary symptoms of GERD – heartburn and regurgitation – are thought to occur because the lower esophagus is exposed to the acid contents of the stomach, predominantly as a result of transient lower esophageal sphincter relaxations (TLESRs) [2]. Current pharmacological treatments for GERD focus on the suppression of gastric acid secretion by the use of proton pump inhibitors (PPIs). Although PPI therapy is effective in most patients with GERD, approximately 20–30% continue to experience reflux symptoms despite PPI treatment [3].

TLESRs are thought to be responsible for about 80% of reflux episodes in patients with GERD, and are therefore a suitable target for treatment [4, 5]. This strategy may be particularly appropriate for patients who have reflux symptoms despite taking a PPI because such symptoms, when not attributed to suboptimal PPI therapy and/or poor PPI treatment adherence, may be elicited by exposure of the esophagus to weakly acid or non-acid reflux [3, 6–8].

The stimulation of γ-aminobutyric acid type B receptors (GABA_B_), both peripherally and centrally, has been shown to inhibit TLESRs [9]. The GABA_B_ agonist baclofen has been used to inhibit TLESRs and reflux episodes, both in healthy individuals and in patients with GERD, but the central effects of tiredness and sleepiness have prevented widespread use of this drug [10–13].

The novel GABA_B_ agonist lesogaberan (AZD3355) significantly inhibits TLESRs and reflux episodes in humans, but has been assessed only as a single 0.8 mg/kg dose and a twice-daily 65 mg dose [14, 15]. The aim of this study was to explore the dose–response effects of lesogaberan, relative to placebo, on reflux variables, and on the pharmacokinetics, and safety and tolerability profiles, in patients with GERD who have symptoms despite PPI therapy.

## Methods

### Study participants

This study was carried out at a single centre in the USA (Oklahoma Foundation for Digestive Research) and all patients gave written, informed consent before entering the study.

Participants were all patients with GERD who had a partial response to PPI treatment. Criteria used to identify this group of men and women were: a history of GERD symptoms of at least 6 months in addition to ongoing symptoms [defined as ≥ 3 days with at least moderate heartburn (burning feeling behind the breastbone) or ≥ 3 days with at least moderate regurgitation (unpleasant movement of material upwards from the stomach)] reported in the 7-day recall Reflux Symptom Questionnaire (RESQ-7) administered within 21 days of study entry; continuous treatment in the 4 weeks before enrolment with daily, optimized and unchanged PPI therapy, with doses according to the US label for any GERD indication (an optimized PPI treatment is a treatment that, according to the investigator’s judgement, cannot be improved further by changing brand or dose regimen); and completion of 8 weeks of treatment with a PPI in patients with reflux esophagitis verified by endoscopy in the 8 weeks before enrolment. Participants were also required to be healthy (other than having GERD) men or women, aged 18–70 years, with a body mass index (BMI) of 18.5–35.0 kg/m^2^, and with clinically normal physical findings and laboratory values at the pre-entry visit. In addition, participants had to have a PPI prescription with refills covering the entire study period, or physician instructions to use equivalent over-the-counter PPI medication.

Patients were excluded if they had shown no improvement in GERD symptoms during PPI therapy. Other exclusion criteria were the concomitant use of drugs that have a narrow therapeutic window (such as warfarin or digoxin) or that could interfere with the pharmacodynamic effects of lesogaberan (such as baclofen or supplements containing GABA), alter gastrointestinal symptoms (such as type 2 histamine receptor agonists) or damage the mucosal lining of the gastrointestinal tract (such as non-steroidal anti-inflammatory drugs, or acetylsalicylic acid in doses > 162 mg/day). Individuals were also excluded if they had clinically significant disorders that could interfere with the study or compromise patients’ safety (e.g. cardiovascular, respiratory, hepatic, renal, metabolic, psychiatric or neurological disorders, or gastrointestinal disorders besides GERD), or had a history of syncope, heart disease, malignant disease, drug addiction or abuse, electrolyte imbalances or severe allergic or hypersensitive reactions. Pregnant or breastfeeding women were also excluded from the study (women of childbearing age were required to use effective contraceptive measures).

### Study drugs, design and ethics

**Figure 1:**
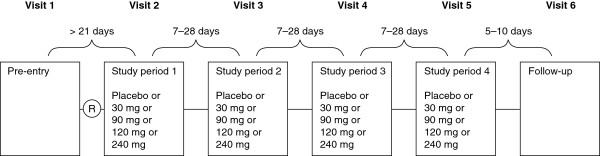
**Study design.** Patients were assigned to four of five treatments by being randomized (R) to one of the following treatment sequences: EABC (n = 2), ABCE (n = 3), BCED (n = 3), CEDB (n = 3), EDBA (n = 3), DABE (n = 3), ABEC (n = 2), BECD (n = 2), ECAD (n = 3) or CADE (n = 3), where A, B, C and D correspond to lesogaberan 30, 90, 120 and 240 mg, respectively, and E corresponds to placebo. The randomization was performed in blocks of consecutive patient numbers.

This was a randomized, double-blind, placebo-controlled, four-way crossover, phase 2a study. An outline of the study design is shown in Figure 1. Patients were randomized to receive one of 10 treatment sequences each consisting of placebo and three of the four different lesogaberan doses (30, 90, 120 and 240 mg). These were given on different days, each separated by a 7–28-day washout period. This washout period far exceeds the likely maximum duration of pharmacological activity, which is conventionally estimated as five times the pharmacological half-life of a drug (which, for lesogaberan, is approximately 11–13 h) [16], meaning that at maximum only 0.01% of the peak concentration (C_max_) remains at the end of the wash-out period. Each dose was given twice over a 24-h period in the form of modified-release capsules: one taken 1 h before breakfast after fasting the night before, and the other taken 1 h before dinner. (Note that patients fasted overnight before each study period: no food after 22:00 and no fluids after 24:00.) Treatment with patients’ usual, regular and optimized dose of PPI was continued throughout the study.

The study (ClinicalTrials.gov identifier: NCT01043185) was designed and performed in accordance with ethical principles originating in the Declaration of Helsinki, and the study was consistent with guidelines of the International Conference on Harmonisation and Good Clinical Practice, regulatory requirements, and the AstraZeneca policy on bioethics and human biological samples. In addition, the University of Oklahoma Health Sciences Centre’s Institutional Review Board approved the study protocol. The investigator obtained signed informed consent from the potential patients, assigned them unique enrolment numbers, determined patient eligibility and assigned eligible patients unique randomization codes. Patient randomization was performed in blocks of consecutive patients using a scheme generated by AstraZeneca R&D, Mölndal, Sweden, using the global randomization system (GRand). All investigators and study personnel were blinded to patient randomization.

The primary objective of the study was to estimate the dose–response effects of lesogaberan, in partial responders taking optimised PPI therapy, on the total number of reflux episodes during a 24-h period (primary variable). Secondary objectives included assessing the effect over 24 hours of the 4 different doses of lesogaberan (AZD3355) compared to placebo, on the number of reflux episodes (total, acid, weakly acid and non-acid), the relationship between reflux episodes and GERD symptoms, the height (proximal extent) and content (liquid, gas) of reflux, and the time with esophageal pH < 4 (esophageal acid exposure) during the 24-h period. The pharmacokinetics [area under the curve (AUC), C_max_ and time to reach C_max_ (t_max_)] and the safety and tolerability profiles of lesogaberan in relation to dose were also assessed as part of the secondary objectives of the study, as was the relationship between the pharmacokinetics and pharmacodynamics of lesogaberan.

### Pharmacodynamic assessments

Impedance–pH measurements were taken for 24 h after the first administration of each dose using a Sleuth® multichannel intraluminal impedance ambulatory system (Sandhill Scientific, Inc., Highlands Ranch, CO, USA). This device includes a data logger, impedance–pH amplifiers, and a catheter consisting of two antimony pH sensors (calibrated before and after recordings were taken using standard pH 7 and pH 1 buffers) placed 5 cm above and 10 cm below the lower esophageal sphincter (LES) and eight impedance electrodes placed 2, 4, 6, 8, 10, 14, 16 and 18 cm above the LES. The electrodes are paired to measure impedance at 3, 5, 7, 9, 15 and 17 cm above the LES. The position of the LES was located by a manometric recording before the study medication or placebo was given.

Impedance–pH readings were analysed by one investigator (JP) using dedicated software (BioVIEW Analysis®, version 5.5.4; Sandhill Scientific Inc.) and were then checked manually. This was performed in a blinded manner and the results were used to determine the number of reflux episodes, defined as a period when impedance decreased to less than 50% of baseline (liquid episode) or increased to more than 150% of baseline (gas episode), propagating aborally from the most distal channel. Acid, weakly acid and non-acid reflux episodes were defined as episodes lasting longer than 5 s with a pH of < 4, 4.0–6.5 and > 6.5, respectively [17]. The decision to use a cautious cut-off pH of 6.5 was taken in order to ensure that no measurements near to a pH of 7 would be erroneously categorized as weakly acidic. These definitions have previously been employed for studies of this type [18]. During the 24-h period of ambulatory impedance–pH measurement, patients used a data logger and a diary card to record their meal intake and periods in supine position, as well as any GERD symptoms.

### Pharmacokinetic and safety assessments

Blood samples were taken 1 h before the first administration of each dose, and at regular intervals during the 24-h period after the first administration of each dose, to assess the concentration of lesogaberan in plasma over time.

Sitting and orthostatic blood pressure and pulse rate were also assessed pre-dose and 2 h after the first dose of lesogaberan or placebo at each treatment visit. Patients ate meals (standardized primarily according to fat content and pH) approximately 1 h after each dose of study medication or placebo, and at the same time points during each study period. Patients were monitored for adverse events from the first dose administration until the follow-up visit 5–10 days after receiving the last dose. The active treatment period for each dose was defined as the time from the first dose of study drug to 24 h after the second dose of study drug in each treatment period. Patients also underwent active questioning to determine whether they experienced syncope, or feeling faint, lightheadedness, dizziness or paraesthesia before study treatment started and after removing the impedance–pH catheter. A 12-lead digital electrocardiogram (ECG) was scheduled at the screening visit, before and 2 h after the first dose of lesogaberan or placebo at each treatment visit, and at the follow-up visit. Blood and urine samples were taken at the pre-entry visit (full laboratory screen), and before and 24 h after the first dose of lesogaberan or placebo at each treatment visit (reduced laboratory screen) to assess clinical chemistry and haematology, and to screen for indications of drug abuse.

### Statistical analysis

The efficacy evaluation of the different doses of lesogaberan was based on data from all patients not affected by major protocol deviations and violations relevant to the analysis of a specific variable. The safety analysis set comprised all patients who received at least one dose of lesogaberan or placebo, and for whom post-dose data were available. The pharmacokinetic analysis set was based on a subset of the safety analysis set and only included patients with no major protocol deviations thought to affect pharmacokinetics significantly.

The sample size was based on data from a previous clinical trial of lesogaberan, conducted by Boeckxstaens and colleagues [14], in which the geometric mean number of total reflux episodes was 30.6 (95% confidence interval [CI] 20.9, 44.7). This corresponded to an approximately two-fold difference between the upper and lower CI limits, which was sufficient to resolve statistically significant differences relative to placebo. The between and within-subject standard deviation in the Boeckxstaens et al. study was 0.79 and 0.25, respectively. This level of variability was incorporated into a statistical model, based on the current study design and a sample size of 20 patients. The resulting simulation predicted 95% CI intervals for the geometric mean number of total reflux episodes (the primary variable) with an approximate two-fold difference between the upper and lower limits. A sample size of 20 patients was therefore deemed sufficient for the current study, based on at least 14 patients being assigned to each of the lesogaberan 30 mg and 240 mg doses, 16 to each of the lesogaberan 90 mg and 120 mg doses, and 20 to placebo. To ensure the minimum number of patients had evaluable data for each dose, 27 patients were randomized.

The effect of each dose of lesogaberan was measured as the proportional difference, relative to placebo, in the geometric mean of the reflux variables assessed over 24 h. The analysis was based on a mixed-effect model with treatment, period and sequence as fixed effects and patient as a random effect. Geometric means and relative differences in geometric means were reported with 95% CIs. A p value of < 0.05 was considered to indicate statistical significance and no corrections for multiple testing were performed. Multiplicity was accounted for by using a step-down sequential procedure comparing lesogaberan doses with placebo, starting at a 240 mg dose and, in the event of rejection of the null hypothesis, comparison of descending doses [19].

The relationship between the pharmacokinetics and pharmacodynamics of lesogaberan was investigated by estimating the exposure–response curve and dose–response curve. This was done using a mixed-effect E_max_ model to explore the mean values of the number of reflux episodes measured for 0–24 h against the dose of lesogaberan used and the mean plasma concentration of lesogaberan observed for each dose.

## Results

### Patient flow, follow-up and baseline characteristics

**Figure 2:**
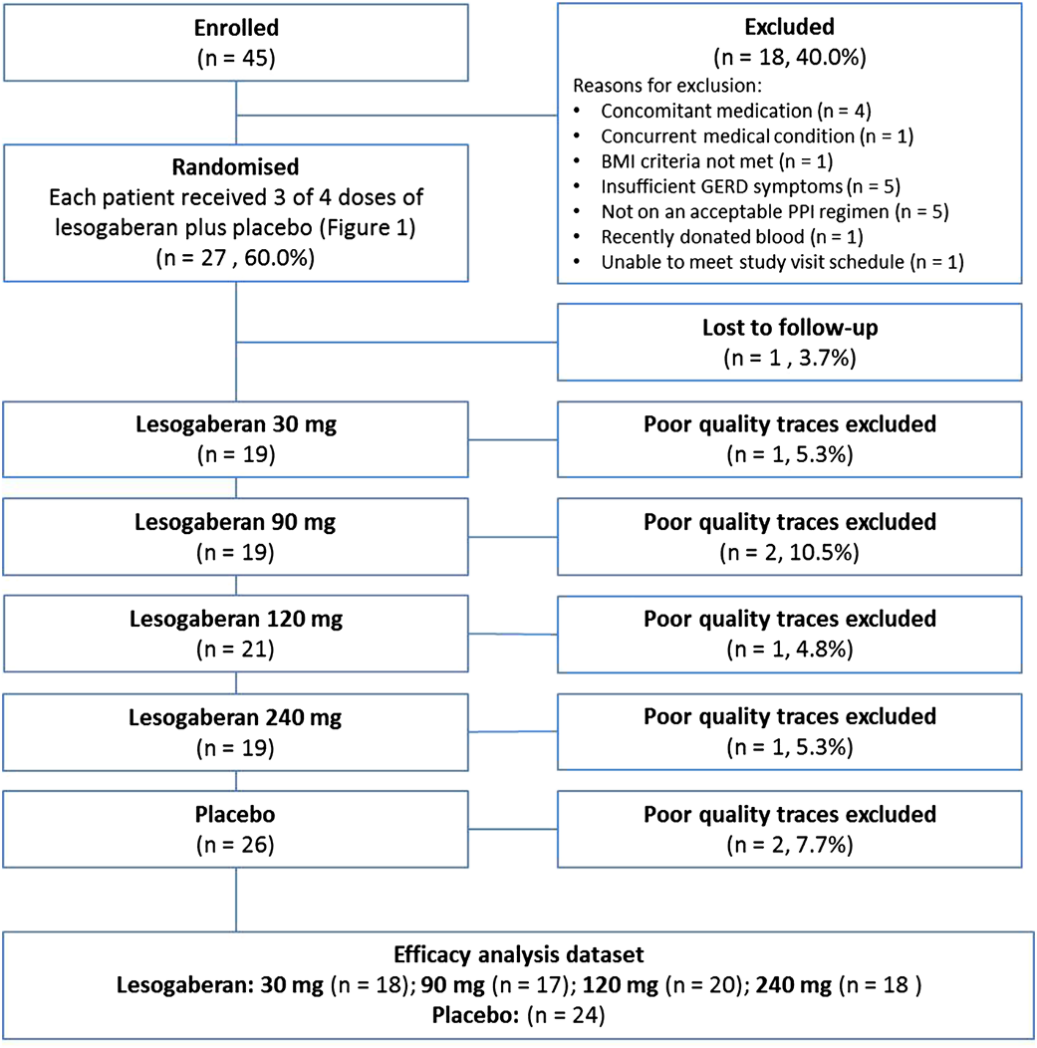
**Study flow.** In total, 27 patients were randomised to receive placebo and 3 of the 4 doses of lesogaberan, based on the randomly assigned treatment sequences shown in Figure 1 (26 patients completed the study). Overall, 7 poor-quality traces were excluded from the final evaluable dataset used to estimate the dose–response effects of lesogaberan (primary outcome).

The first patient was enrolled on 17 December 2009 and the last patient visit occurred on 7 May 2010. To assess eligibility for the study, 45 patients were pre-screened by telephone. Subsequently, 18 patients were excluded (four were on exclusionary concomitant medications; one had an exclusionary concurrent medical condition; one did not meet the BMI criteria; five did not have enough breakthrough GERD symptoms; five were not on an acceptable PPI regimen; one had recently donated blood; one could not meet the study visit schedule demands Figure 2). In total, 27 participants were therefore randomized to receive study medication, with a mean age of 43 years (range: 18–68 years) and a mean BMI of 30 kg/m^2^ (range: 23–34 kg/m^2^). Of these, 52% (14/27 patients) were male; 93% (25/27 patients) were white; and 11% (3/27 patients) were current smokers. Current optimized PPI therapy consisted of esomeprazole 20 mg (2 patients), esomeprazole 40 mg (2 patients), lansoprazole 15 mg (2 patients), omeprazole 20 mg (18 patients), omeprazole 40 mg (1 patient) and pantoprazole 40 mg (2 patients). One patient was subsequently lost to follow-up after testing positive for drug abuse. The pH–impedance data obtained from some of the treatment arms for three patients were excluded from the efficacy analysis because of poor quality tracings (Figure 2). Within the efficacy analysis set, 24 patients received placebo, and the numbers in each lesogaberan dose group were: 30 mg, n = 18; 90 mg, n = 17; 120 mg, n = 20; 240 mg, n = 18. Overall, very few symptoms were reported across the treatment groups (median of 0 symptoms during dosing with lesogaberan 120 mg, 0.5 symptoms for lesogaberan 240 mg, and 1.0 symptom for lesogaberan 30 mg and 90 mg and for placebo. Meaningful statistical analyses were therefore not possible for these data.

Both the pharmacokinetic and safety analysis sets consisted of all 27 randomized patients. Patients entering this study had, on average, 163 months’ history of reflux symptoms (range: 12–360 months). No patients had a history of hiatal hernia, one patient had a history of erosive esophagitis, and seven (26%) tested positive for *Helicobacter pylori* infection.

### Efficacy (pharmacodynamic) results

#### Number of reflux episodes

**Figure 3:**
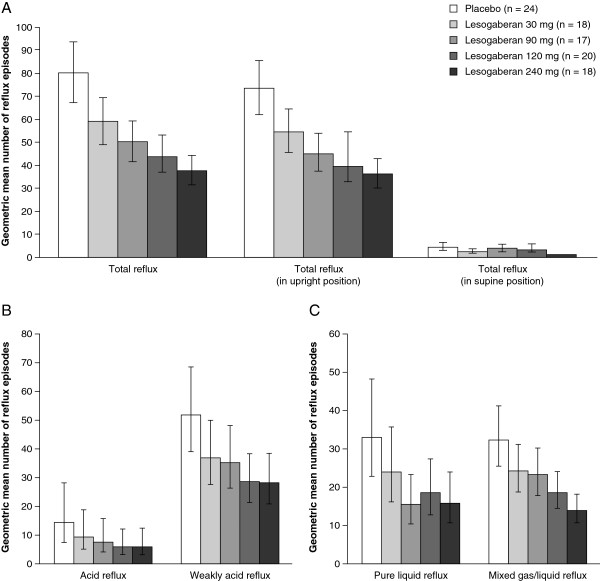
**Observed effects (efficacy analysis set) of lesogaberan 30, 90, 120 and 240 mg compared with placebo on: (A) the total number of reflux episodes; (B) the number of acid and weakly acid reflux episodes; and (C) the number of pure liquid and mixed liquid/gas reflux episodes.** Data are presented as geometric means with 95% confidence intervals.

During treatment with all four doses of lesogaberan, the mean total number of reflux episodes was significantly reduced relative to periods during which placebo was given (Table 1; all p < 0.001). The magnitude of the effect of lesogaberan on the number of reflux episodes was dose-dependent (Figure 3A), with a reduction in the mean number of reflux episodes of 26.2% relative to placebo in patients receiving the 30 mg dose, compared with a mean reduction of 52.8% in patients receiving the 240 mg dose. Most reflux episodes occurred while patients were in an upright position and the dose-dependent effects of lesogaberan were more apparent for this type of reflux (Figure 3A). For patients in a supine position, the only statistically significant reduction relative to placebo in the mean number of reflux episodes was observed for the highest dose of lesogaberan (69.1%; p < 0.0001; Table 1). Data on reflux variables obtained from patients while in a supine or upright position are combined from this point onwards.

**Table 1:** **Pharmacodynamic effects of lesogaberan 30, 90, 120 and 240 mg relative to placebo (efficacy analysis set; n = 25)**

Lesogaberan dose (mg)	Difference in geometric mean (%)	95% CI	p value*
**Total number of reflux episodes†**			
30	-26.2	-12.7 to -37.6	0.0006
90	-37.0	-25.0 to -47.1	< 0.0001
120	-45.0	-35.5 to -53.1	< 0.0001
240	-52.8	-44.3 to -60.0	< 0.0001
**Number of reflux episodes in upright position**			
30	-25.2	-11.1 to -37.1	0.0013
90	-38.4	-26.3 to -48.5	< 0.0001
120	-46.1	-36.5 to -54.2	< 0.0001
240	-50.7	-41.5 to -58.4	< 0.0001
**Number of reflux episodes in supine position**			
30	-37.3	+2.0 to -61.5	0.0597
90	-12.3	+45.2 to -47.0	0.6042
120	-31.2	+9.3 to -56.7	0.1117
240	-69.1	-50.0 to -80.9	< 0.0001
**Number of acid reflux episodes†**			
30	-34.7	+3.2 to -58.6	0.0677
90	-45.5	-12.3 to -66.1	0.0132
120	-57.9	-35.0 to -72.7	0.0002
240	-57.1	-32.5 to -72.7	0.0004
**Number of weakly acid reflux episodes†**			
30	-28.3	-7.5 to -44.5	0.0113
90	-31.4	-10.7 to -47.4	0.0059
120	-44.5	-29.2 to -56.4	< 0.0001
240	-45.1	-29.3 to -57.3	< 0.0001
**Number of pure liquid reflux episodes†**			
30	-27.9	+0.6 to -48.3	0.0540
90	-53.0	-33.6 to -66.7	< 0.0001
120	-44.1	-23.3 to -59.2	0.0005
240	-51.9	-33.1 to -65.4	< 0.0001
**Number of mixed gas/liquid reflux episodes†**			
30	-25.0	-5.1 to -40.7	0.0175
90	-28.3	-8.4 to -43.8	0.0084
120	-42.5	-28.1 to -54.0	< 0.0001
240	-56.7	-45.4 to -65.7	< 0.0001
**Proximal extent of reflux (cm)†**			
30	-39.1	-4.5 to -61.2	0.0312
90	-58.9	-34.4 to -74.2	0.0003
120	-66.3	-48.3 to -78.0	< 0.0001
240	-63.3	-42.8 to -76.5	< 0.0001
**Proportion of time with esophageal pH < 4,%†**			
30	-69.8	-27.3 to -87.4	0.0083
90	-56.1	+9.0 to -82.3	0.0752
120	-65.7	-21.1 to -85.1	0.0126
240	-73.0	-35.6 to -88.6	0.0037
**Proportion of time with intragastric pH < 4,%†**			
30	+40.4	+10.3 to +78.8	0.0066
90	+23.7	-3.8 to +58.9	0.0957
120	+3.6	-17.7 to +30.3	0.7625
240	+27.7	+0.6 to +62.1	0.0451

CI, confidence interval.

*Based on a mixed-effect analysis of variance model for log-transformed data.

†In upright or supine position.

Lesogaberan reduced the mean number of acid and weakly acid reflux episodes in a dose-dependent manner (Figure 3B), with the only non-significant decrease occurring for lesogaberan 30 mg in relation to acid reflux (p = 0.068; Table 1). All four doses of lesogaberan significantly reduced the mean number of mixed gas/liquid reflux episodes relative to placebo (Table 1; all p < 0.05), and these effects also appeared to be dependent on the dose of lesogaberan (Figure 3C). A dose-dependent effect for lesogaberan was less clear for pure liquid reflux (Figure 3C), with similar reductions of 53.0%, 44.1% and 51.9% observed for lesogaberan 90, 120 and 240 mg, respectively (Table 1). However, the smallest reduction in the mean number of pure liquid reflux episodes relative to placebo was observed for lesogaberan 30 mg, and this was the only dose that did not significantly reduce this type of reflux (p = 0.0540; Table 1). No significant effect was detected for lesogaberan relative to placebo in terms of the mean number of pure gas reflux episodes or the mean number of non-acid reflux episodes (data not shown).

#### Other reflux characteristics

**Figure 4:**
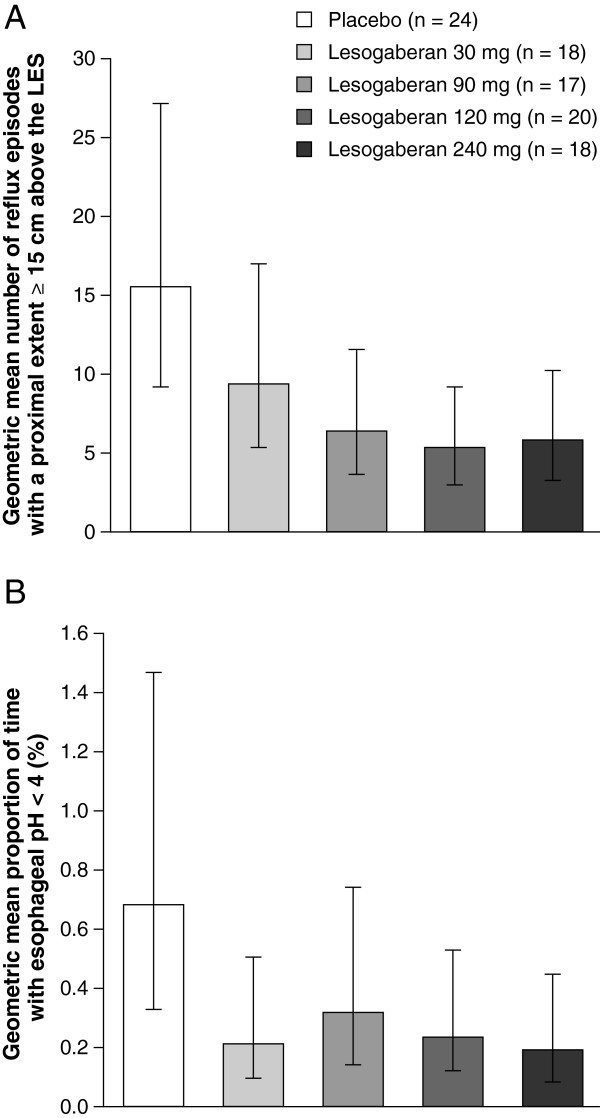
**Observed effects (efficacy analysis set) of lesogaberan 30, 90, 120 and 240 mg compared with placebo on: (A) the proximal extent of reflux; and (B) the proportion of time with esophageal pH < 4.** Data are presented as geometric means with 95% confidence intervals.

Relative to placebo, all four doses of lesogaberan significantly reduced the mean number of reflux episodes that had a proximal extent at least 15 cm above the LES (Table 1; all p < 0.05). Lesogaberan 90, 120 and 240 mg reduced the proximal extent of reflux to a similar extent relative to placebo (Table 1 and Figure 4A). Three of the four doses (30, 120 and 240 mg) of lesogaberan significantly reduced esophageal acid exposure relative to placebo (all p < 0.05), but this did not appear to occur in a dose-dependent manner (Table 1 and Figure 4B). Intragastric acid exposure was significantly increased relative to placebo in patients receiving lesogaberan 30 mg and 240 mg (Table 1; both p < 0.05).

#### Pharmacokinetic results

**Figure 5:**
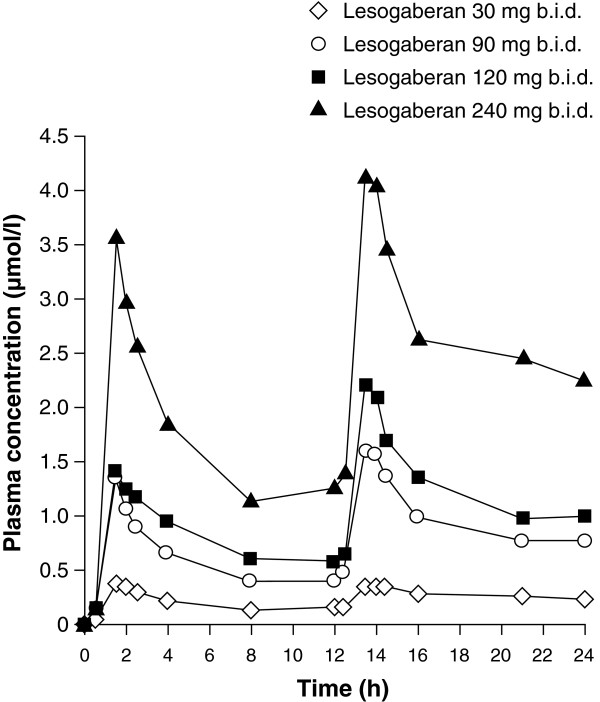
**Arithmetic mean plasma concentration of lesogaberan.** Lesogaberan was administered as doses of 30, 90, 120 or 240 mg twice daily (b.i.d.) over a period of 24 h (pharmacokinetics analysis set).

**Figure 6:**
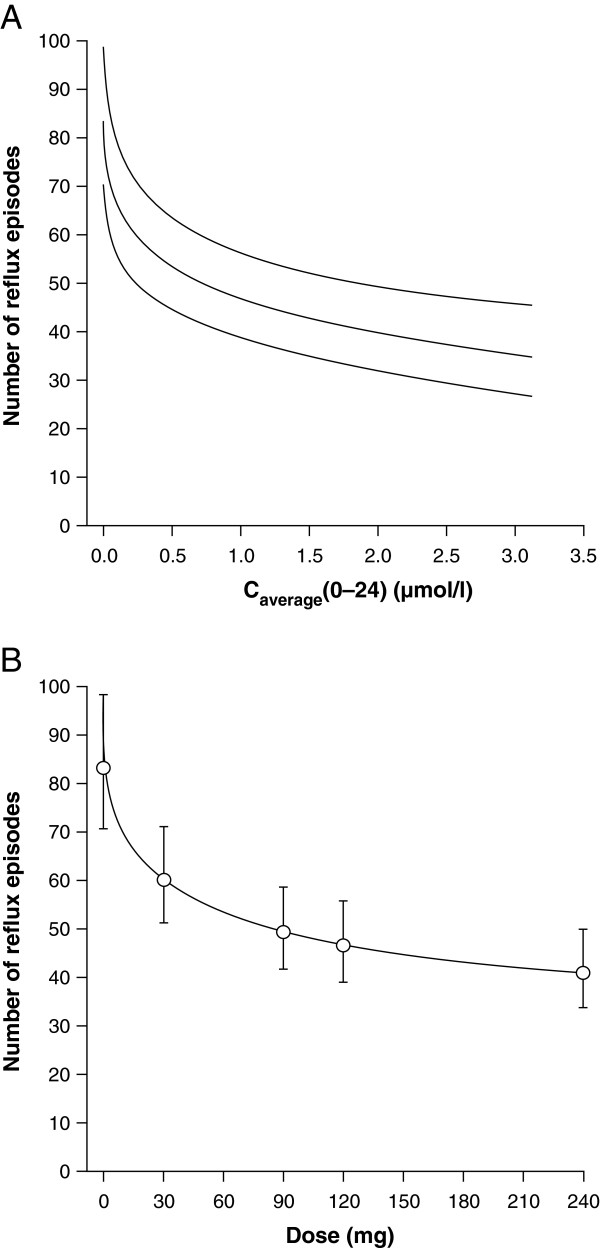
**Number of reflux episodes over a period of 24 h.** Estimation using a mixed-effect E_max_ model of **(A)**, the predicted exposure–response curve with 95% CI curves (intersection of efficacy and pharmacokinetics analysis set) and **(B)**, the dose–response curve with 95% CIs (efficacy analysis set) for lesogaberan. CI, confidence interval.

For all four doses of lesogaberan, absorption from the bloodstream was rapid (Table 2; geometric mean t_max_ 1.7–2.6 h) and plasma concentrations decreased at a similar rate for each dose in the 12-h periods that followed each administration (Figure 5). Exposure to lesogaberan, as assessed by AUC and C_max_ values, varied in proportion to the doses of lesogaberan that were administered (Table 2). A relationship was observed between the number of reflux episodes and the level of exposure to lesogaberan (Figure 6A) and the dose of lesogaberan (Figure 6B).

**Table 2:** **Pharmacokinetic measures in patients during dosing with lesogaberan 30 (n = 18), 90 (n = 20), 120 (n = 21) and 240 mg (n = 19)**

Parameter	Lesogaberan dose (mg)	Geometric mean	Coefficient of variation (%)
**AUC**_**(0–24 h)**_, **μmol · h/l**	30	5.25	46.4
	90	17.3	31.5
	120	22.2	44.8
	240	46.9	50.0
**C**_**max**_ **morning, μmol/l**	30	0.35	59.2
	90	1.30	38.2
	120	1.48	53.5
	240	3.31	69.3
**C**_**max**_ **evening, μmol/l**	30	0.42	59.5
	90	1.68	43.8
	120	2.21	50.3
	240	4.18	36.8
**t**_**max**_ **morning, h**	30	1.7	25.2
	90	1.7	26.7
	120	1.9	53.1
	240	1.7	27.6
**t**_**max**_ **evening, h**	30	2.2	56.4
	90	1.9	47.1
	120	1.9	35.9
	240	2.6	73.4

AUC, area under the curve; C_max_, peak concentration; t_max_, time to reach peak concentration.

### Safety and tolerability assessments

During active dosing, one patient reported headache and nausea while taking lesogaberan 90 mg and one had viral gastroenteritis while taking placebo. No serious adverse events were reported during active dosing periods and no adverse events resulted in discontinuation. One serious adverse event (reversible elevated blood creatinine, alanine aminotransferase and aspartate aminotransferase levels following recent heavy exercise) was detected at the follow-up visit (i.e. not during active dosing) in one patient, but this was not deemed by the investigator to be related to study drug. Slight increases in pulse rate and slight decreases in blood pressure were observed in patients taking lesogaberan compared with those taking placebo, in line with results from previous studies. Orthostatic reactions occurred in three patients, but none had any clinical symptoms and no cardiovascular adverse events occurred. No clinically significant changes in ECG readings were observed.

## Discussion

The primary objective of this study in patients with a partial response to PPI treatment who were taking optimised PPI therapy was to assess the effects of different doses of lesogaberan (given twice daily on a single day), relative to placebo, on the total number of reflux episodes during a 24-h period.

The dose range of lesogaberan that was used in this study was comprehensive in that it encompassed doses both higher and lower than the 65 mg dose used in previous studies in patients with GERD and in patients with GERD who had persistent reflux symptoms despite PPI treatment [14, 20], as well as including a maximum therapeutic dose of 240 mg. The double-blind, crossover design chosen for this study minimized bias and variability, and therefore the sample size needed was reduced. The use of treatment and placebo groups, with patients maintaining their PPI medication throughout the study, allowed the dose-dependent effects of lesogaberan over and above those of PPI therapy alone to be determined. Potential limitations of crossover studies include the possibility of carryover and sequence effects. While such effects can never be completely excluded, they were minimised by the large washout period used between treatments and by randomisation of the treatment sequences. Another limitation was the number of poor-quality traces obtained, although the sample size used was sufficient to ensure that the minimum evaluable data required to achieve adequate statistical power were obtained. Issues of multiplicity, which are a potential limitation when a high number of treatment comparisons are made, were addressed using a step-down sequential procedure comparing lesogaberan doses with placebo, starting at a 240 mg dose and, in the event of rejection of the null hypothesis, comparison of descending doses Finally, statistically significant reductions in reflux episodes do not necessarily equate to clinically relevant reductions in patients’ symptoms, as discussed in further detail below.

Lesogaberan 65 mg (twice daily) has previously been shown to reduce the mean number of total reflux episodes by approximately 35% relative to placebo in patients with GERD who have a partial response to PPI therapy [14]. This finding is consistent with the results of the current study, with lesogaberan 30 mg and 90 mg reducing the mean number of total reflux episodes by approximately 26% and approximately 37%, respectively. The greatest reduction in the mean number of total reflux episodes of approximately 53% was observed in patients receiving lesogaberan 240 mg; dose–response curves indicate that this is close to the maximum effect achievable for this drug (Figure 6B). A similar maximum effect in terms of TLESR reduction was observed in dogs in preclinical studies using lesogaberan at concentrations at which it has a strictly peripheral mode of action [21]. These results indicate that preclinical animal models can be used successfully to predict the clinical effects of novel therapeutics that target mechanisms of TLESR generation.

Lesogaberan 65 mg (twice daily) given as an add-on therapy to PPIs in patients with a partial response to PPI treatment has also been shown in a phase 2a study to significantly increase the proportion of patients whose reflux symptoms respond to treatment (response defined as a maximum of one 24-h period with heartburn and/or regurgitation of not more than mild intensity during the last 7 treatment days), although the proportion of responders was small (8% for placebo vs. 16% for lesogaberan) [20]. The results of the current study suggest that this effect could be improved using a higher dose of lesogaberan. Nevertheless, a subsequent phase 2b study has found that lesogaberan 240 mg does not have a clinically important effect on GERD symptoms in partial responders to PPI treatment [22] which, in combination with some potential safety signals (reversible elevated alanine transaminase levels >5 times the upper limit of normal in 6 patients), led to the discontinuation of the development of lesogaberan in 2012. One reason why the effects of lesogaberan on TLESRs and acid exposure did not translate into significant symptom relief could be the presence of patients with functional heartburn in the study population, which is likely given that symptom criteria were used for the selection of patients, rather than objective measures of GERD such as pH-metry. These data show strong pharmacophysiological evidence for targeting of the lower esophageal sphincter to reduce reflux events. The fact that this reduction in reflux events does not correlate with a reduction in symptom reporting in the current study does not devalue the physiological appropriateness of this mechanism; instead, it highlights that our understanding of the relationship between reflux events and patient-reported symptoms is still lacking [23].

An unexpected finding of the current study was that lesogaberan significantly increased the percentage of time with an intragastric pH < 4, despite decreasing the number of acid reflux episodes and the percentage of time with an esophageal pH < 4. It is unclear why this occurred in the current study, especially given that the effect was highly variable, with significant increases in gastric acidity only occurring at the lowest and highest doses of lesogaberan. It is, however, worth noting that the number of acid reflux episodes and percentage of time with an esophageal pH < 4 could still be reduced under these conditions, as long as reflux frequency (i.e. the frequency at which stomach contents actually reach the esophagus) and/or volume is reduced enough to counteract any increase in gastric acidity. It must be noted that the placebo group reported few symptoms (median of 1.0 symptom) during the 24-h study period. These patients may have adhered better to their treatment during the study. However, the paucity of symptom data did not allow us either to compare symptoms between groups, or to relate symptoms to reflux inhibition.

## Conclusions

In general, the results of this study were very clear, with lesogaberan reducing most measures of reflux in a dose-dependent manner, and the majority of these effects being significant relative to placebo in partial responders taking optimised PPI therapy. The dose-dependent effects of lesogaberan were consistent with the patients’ level of exposure to this drug, with pharmacokinetic measures such as the maximum AUC for plasma concentration and C_max_ varying in proportion to the dose of lesogaberan. Moreover, all doses of lesogaberan appeared to be associated with a good tolerability profile and did not cause any serious or clinically relevant adverse events. In conclusion, this study confirms that lesogaberan works as a reflux inhibitor in a predictable, dose-dependent way.

## Abbreviations

AUC: Area under the curve
BMI: Body mass index
CI: Confidence interval
C_max_: Peak concentration
ECG: Electrocardiogram
GABA_B_: γ-aminobutyric acid type B receptor
GERD: Gastroesophageal reflux disease
LES: Lower esophageal sphincter
PPI: Proton pump inhibitor
RESQ-7: 7-day recall Reflux Symptom Questionnaire
TLESR: Transient lower esophageal sphincter relaxation
t_max_: time to reach C_max_.
